# Improving medical missions: A student and trainee tool for ethical engagement

**DOI:** 10.1371/journal.pgph.0007415

**Published:** 2026-10-15

**Authors:** Issa Beatriz Palomata, Joshua D. Guzman, Britney Momanyi, Kala M. Mehta

**Affiliations:** 1 San Francisco State University, San Francisco, California, United States of America; 2 Cornell University, Ithaca, New York, United States of America; 3 STRONG Lab, Department of Epidemiology and Biostatistics, University of California, San Francisco, California, United States of America; PLOS: Public Library of Science, UNITED STATES OF AMERICA

## Introduction

Medical missions have long been regarded as acts of compassion, with health professionals traveling to resource-limited settings to provide care where it is urgently needed [1,2]. Medical missions are also known by other names such as medical outreach, or medical service. These trips often provide a meaningful difference, sometimes representing the only opportunity to access medical attention, essential medications, or surgical care [3]. Yet without careful planning, cultural humility, and long-term follow-up, medical missions can unintentionally cause harm [1,2]. Temporary relief may be delivered while local health systems remain under-resourced or increasingly reliant on external aid, and short-term interventions can disrupt existing care pathways rather than strengthening them [4,5]. These outcomes typically reflect structural limitations in how missions are designed and evaluated, not malicious intent. Our aim here is not to critique medical missions as a model of care, but to strengthen the experience for patients, host communities, and the students who participate in them. Much of this risk plays out through premedical students and trainees, who often encounter global health through volunteer missions before any formal training in clinical ethics or professional accountability.

Prior literature on medical missions has focused on institutional practices, sustainability, and short-term impacts on host health systems, alongside a substantial body of ethical guidance and best-practice frameworks for global health engagement [3,6]. Far less attention has been given to premedical students and trainees specifically [7–9]. This article's contribution is therefore not a new set of ethical principles, as established frameworks already exist [3,6], but their application to the premedical and trainee stage: medical missions function as sites of early professional socialization, making this stage a critical point for ethical intervention before formal medical training begins. Some programs already provide pre-departure ethics training; the tool proposed here is meant to complement such efforts and offer a starting point when students do not have access to other frameworks [9,10].

Premedical students occupy a distinctive position between observation and participation, supplying needed “person power” while absorbing implicit norms about scope of practice and professional competence through direct experience rather than instruction. The section that follows draws on the firsthand experience of I.B.P. (Issa Beatriz Palomata) during a 2024 medical mission in Bayambang, Pangasinan Province, Philippines, to illustrate how these tensions inform the reflection tool.

## A tool for ethical reflection

If missions function as early sites of professional socialization, ethical reflection must be structured: introduced before departure, applied during service, and revisited upon return [1,11]. The proposed reflection tool (Table 1) provides structured prompts for use before departure, during service, and after return.

**Table 1:** Pre-Departure, During, and Post-Mission Reflection Tool for Premedical Students and Trainees.

Ethical Consideration	Before Departure	During Mission	After Return
Reflect on resource burden	• What is your primary role on this mission? • Who supervises and orients you, and what does that take from their time? • In what ways might your presence reduce strain on local staff? In what ways might it add to their load?	• Is your presence reducing or adding to the workload of local staff — and how do you know? • Have you observed moments where your supervision or orientation diverted clinical attention? • What adjustments, if any, have you made to reduce your resource burden on the host team?	• Looking back, did your role reduce strain on the host team or add to it? • What surprised you about how your presence affected local staff and patients? • What would you change about your preparation or conduct to reduce resource burden next time?
Prioritize contribution over learning	• What can you meaningfully contribute that the team actually needs? • Where does your educational interest risk overshadowing community benefit? • If your learning came at a cost to a patient or staff member, what would that look like?	• In practice, where have learning and contribution aligned — and where have they been in tension? • Have you caught yourself prioritizing an experience for your own development over the team's needs? • Has there been a moment where you stepped back from a task because it served you more than the patient?	• When did learning and contribution align, and when did they come into tension? • Did anything you did serve your résumé more than the community? • What would ethical contribution look like differently on a future mission?
Understand the local health system	• How is healthcare typically financed and delivered in the host community? • What follow-up infrastructure exists for patients seen during the mission? • Who are the local providers, and how do they describe the gaps your team may fill?	• What have you learned about the local health system that you did not know before arrival? • Have you observed gaps in follow-up or continuity of care? What do they look like in practice? • How are local providers navigating or responding to the mission team's presence?	• What about the local health system did you misunderstand or underestimate before arriving? • How will patients seen during the mission access ongoing care once your team departs? • How has this experience changed how you think about short-term versus long-term health system engagement?
Clarify scope of practice and supervision	• What tasks am I qualified to perform under supervision in my home setting? • What tasks would I refuse to perform at home, and why? • Who do I ask if I am uncertain whether a task is within my scope?	• Have you been asked or expected to perform any task that exceeds your training or home-institution scope? • How have you handled uncertainty about scope — and was that response consistent with what you planned? • Have you observed differing standards of care between visiting and local teams?	• Was I asked or expected to perform any task that exceeded my training or home-institution scope? • How did I respond, and what would I do differently next time? • What does geographic variability in standards of practice mean for your own professional development?
Maintain personal health and preparedness	• What vaccinations, prophylaxis, and travel preparation do I need? • What are my plans for sleep, hydration, food safety, and mental health during the mission? • Who is my emergency contact, and what is the local health-seeking plan if I become ill?	• Are you maintaining the health and self-care habits you planned? Where have you fallen short? • Have any illness, fatigue, or mental health challenges affected your ability to contribute? • Are you monitoring your teammates’ wellbeing as well as your own?	• Did I take adequate care of my health to remain a reliable and useful team member? • What would I prepare or do differently to protect both my wellbeing and the host team's resources? • Did any health or wellness challenges affect the care or support you were able to provide?
Support longitudinal engagement	• Is this mission part of an ongoing relationship with the host community? How long has it existed? • What forms of engagement continue between missions? • How do local partners describe the value of long-term relationship versus episodic visits?	• What evidence of long-term partnership — or its absence — do you observe on the ground? • Are local providers and community members describing needs that go beyond what this mission can address? • What relationships are you building that could be sustained after departure?	• How can I personally contribute to continuity, beyond this trip? • Are there relationships I should sustain — with local providers, patients’ families, or organizers? • What would it look like for this mission to evolve toward a more sustained partnership model?
Remain accountable after departure	• What forms of follow-up am I committing to before I leave (advocacy, fundraising, communication)? • What would it look like to hold myself accountable to those commitments six months from now? • Who will hold you accountable — a mentor, peer, or supervisor?	• Based on what you are seeing, what specific follow-up actions feel most important? • Are there patient stories or system gaps you want to document for advocacy upon return? • What commitments are you making — formally or informally — to people you are meeting here?	• What commitments did I make, and have I kept them? • What concrete actions will I take in the next month to follow through? • How will I remain engaged with global health in a way that is accountable, not just aspirational?
Engage in structured reflection	• Who will I discuss this experience with after I return (mentor, faculty, peer)? • What questions do I most want to think through during and after the mission? • What would I want a future premedical participant to know before they depart?	• What ethical question or tension has surfaced that you did not anticipate before departure? • Are you making time — even briefly — to reflect, write, or discuss what you are experiencing? • Has anything happened that you want to make sure you revisit carefully upon return?	• What have I changed my mind about? • What ethical question remains unresolved for me, and how will I keep working on it? • How has this experience shaped how you understand your role and responsibility in global health?

During the mission, I.B.P. assisted with medication distribution, translation, and minor procedures in gymnasiums converted into temporary clinics, where hundreds of patients were screened for hypertension and diabetes or treated for conditions left unaddressed for months or years [12,13]. These examples map directly onto the tool's principles. Translating for a woman with markedly elevated, previously unchecked blood pressure, I.B.P. helped translate the patient report to the clinician who initiated treatment, an interaction that may have been the patient’s only cardiovascular screening, with no clear mechanism for reassessment once the team departed [14]. This illustrates the tension between resource burden and continuity of care: used before departure, the tool might prompt a student to weigh whether a role like translation truly reduces burden on local staff; used after return, it invites students to look back and ask whether that anticipated benefit held up in practice. Some patients were turned away once supplies or time ran out, while tasks restricted for students at home occasionally became informally permissible abroad [5,12], illustrating the importance of the tool's prompts on scope of practice and on accountability.

This tool is not intended to replace existing curricula. Some institutions already provide structured pre-departure orientation or post-mission debriefing [9,10]; this instrument is intended primarily for programs without a comparable framework, and as a prototype for future empirical evaluation rather than a validated intervention. Supervising clinicians retain formal responsibility for patient care; the tool instead shapes how premedical students come to understand equity, accountability, and professional boundaries as they enter the field.

## Accountability and follow-up

Ethical engagement in medical missions requires accountability at both the institutional and the student level.

Students are responsible for using the reflection tool honestly; remaining within the scope-of-practice boundaries that would apply at home; prioritizing meaningful contribution over résumé-building or personal educational gain; and reflecting critically before, during, and after participation [3,5].

Universities and mission organizations are responsible for a broader set of obligations: conducting community needs assessments in partnership with local health authorities; involving local healthcare professionals and community representatives in planning and oversight; providing mandatory pre-departure training in cultural humility, local health systems, and language accessibility; establishing feedback channels with host communities and providers; and maintaining post-mission follow-up mechanisms [4,7,9,10]. Program directors can additionally draw on aggregate, de-identified themes from student reflections as one input into these review processes, without repurposing the tool itself as an institutional compliance instrument.

Longstanding institutional partnerships are also what allow missions to avoid becoming extractive. They are built on reciprocity, requiring sustained, ongoing dialogue and partnership rather than one-off service delivery. This includes shared decision-making with local providers and structured opportunities for host communities to shape future visits [5,10]. A listen-first model, in which visiting clinicians work alongside local providers through co-management and mentorship, supports local capacity building rather than bypassing local health workers [9,15]. Students are a pivotal part of this process as they have not yet been shaped by formal training and can listen with compassion and understanding. Framed this way, a mission's goal is to strengthen the local health system, not merely to deliver services to it.

## Conclusion

Medical missions occupy a complicated space in global health: they can deliver essential care in underserved settings while also functioning as early environments in which future physicians begin to learn the art of medicine, about standards of care, as well as the professional boundaries that are considered acceptable. Improving medical missions therefore requires more than institutional redesign; it requires recognizing that professional identity formation begins early, before formal medical training. The tool introduced in Table 1 offers one concrete mechanism for supporting ethical student engagement. We would encourage students to engage with the tool before departure and revisit it after return. In doing so, it creates structured space for critical reflection, while institutions retain responsibility for the structural conditions of the mission itself. Reflecting back, the registration line stretching beyond the gymnasium entrance in Pangasinan, patients turned away once supplies ran out, was not only a scene of unmet need but an early lesson in what it means to take responsibility for the limits of the care medical professionals provide. Student reflection and institutional accountability must work together if that lesson is to translate into lasting change.
